# Optical Frequency Upconversion Technique for Transmission of Wireless MIMO-Type Signals over Optical Fiber

**DOI:** 10.1155/2014/170471

**Published:** 2014-03-16

**Authors:** R. Q. Shaddad, A. B. Mohammad, S. A. Al-Gailani, A. M. Al-Hetar

**Affiliations:** ^1^Lightwave Communications Research Group, Infocomm Research Alliance, Universiti Teknologi Malaysia (UTM), 81310 Johor, Malaysia; ^2^Communication and Computer Engineering Department, Faculty of Engineering and Information Technology, Taiz University, Taiz, Yemen; ^3^Industrial Technical Institute, Mallaa, Aden, Yemen

## Abstract

The optical fiber is well adapted to pass multiple wireless signals having different carrier frequencies by using radio-over-fiber (ROF) technique. However, multiple wireless signals which have the same carrier frequency cannot propagate over a single optical fiber, such as wireless multi-input multi-output (MIMO) signals feeding multiple antennas in the fiber wireless (FiWi) system. A novel optical frequency upconversion (OFU) technique is proposed to solve this problem. In this paper, the novel OFU approach is used to transmit three wireless MIMO signals over a 20 km standard single mode fiber (SMF). The OFU technique exploits one optical source to produce multiple wavelengths by delivering it to a LiNbO_3_ external optical modulator. The wireless MIMO signals are then modulated by LiNbO_3_ optical intensity modulators separately using the generated optical carriers from the OFU process. These modulators use the optical single-sideband with carrier (OSSB+C) modulation scheme to optimize the system performance against the fiber dispersion effect. Each wireless MIMO signal is with a 2.4 GHz or 5 GHz carrier frequency, 1 Gb/s data rate, and 16-quadrature amplitude modulation (QAM). The crosstalk between the wireless MIMO signals is highly suppressed, since each wireless MIMO signal is carried on a specific optical wavelength.

## 1. Introduction

Next generation access networks are planned to provide customers with high data rate, broadband multiple services, and flexible communication. There is strong competition between optical access technologies and wireless access technologies to achieve these requirements, since the bandwidth demand of the end-users has become larger nowadays [1]. The optical fiber access networks provide high-bandwidth digital services and long-distance communication, but they are less ubiquitous. The wireless access networks provide flexible and ubiquitous communication with a low deployment cost. However, its deployment scalability is limited by spectrum and range [2, 3]. The FiWi access network is powerful hybrid architecture of optical backhaul and wireless front-end. This hybrid FiWi access network supports high data rates and throughput with minimal time delay [4].

**Figure 1:**
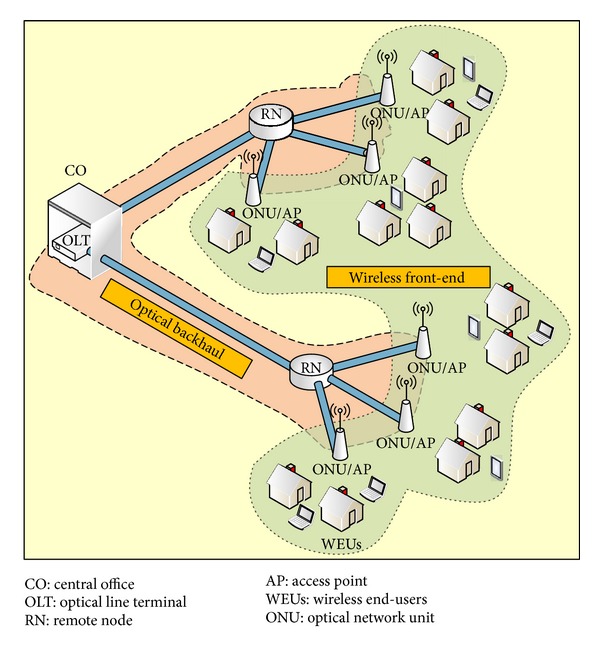
FiWi access network architecture.

Figure 1 shows architecture of a FiWi access network. The optical backhaul is a tree network connecting the central office (CO) and wireless front-end. The optical backhaul is comprised of an optical line terminal (OLT) at the CO, an SMF, a remote node (RN), and multiple access points (APs). The wireless front-end consists of widespread APs to penetrate numerous wireless end users (WEUs). There are two main methods to transmit the wireless signals over the FiWi systems: ROF transmission and digitized radio-over-fiber (DROF) transmission [5, 6].

For wireless broadband transmission, the MIMO radio system has been defined as multiple transmit/receive antennas. The MIMO system is designed to improve transmission range/reliability and deliver higher data transmission rates than the single-input single-output (SISO) system. The wireless MIMO signals are transmitted over fiber to get a powerful integrated FiWi system. The optical fiber is well adapted to pass multiple wireless signals having different carrier frequencies by using ROF technique. However, multiple wireless signals which have the same carrier frequency cannot propagate over a single optical fiber, such as MIMO signals feeding multiple antennas in the FiWi system. The problem starts once multiple MIMO signals are combined and then upconverted by a single optical carrier. Individual MIMO signals could not be separated and recovered thereafter with regular electrical filtering. The simple approach to solve this problem is by transporting each MIMO signal over individual optical fiber. However, this approach will not be cost-effective when many MIMO signals are transmitted over several optical fibers. An approach to solve this problem by using wavelength division multiplexing (WDM) and subcarriers multiplexing (SCM) techniques has also been proposed [7, 8]. These techniques are not cost-effective, since multiple optical sources and photodetectors are required. When SCM technique is used, all except one of the MIMO radio signals are translated to different frequency bands to transport them over fiber. Many frequency converters are then used to translate the delivered signal back to the original frequency band. So the cost and complexity are high in this approach, especially when the number of MIMO signals is large.

The phase quadrature double-sideband frequency-translation technique has been proposed to transport MIMO radio signals over single optical fiber [9]. The achieved modulation symbol rate was limited because the phase and amplitude of the double sidebands were not sufficiently matched due to the dispersion and frequency response of the overall system [9].

Transmission of three MIMO radio signals all with 2.44 GHz carrier frequency over an optical fiber was proposed and demonstrated using an electrical single-sideband frequency translation (ESSB-FT) technique [10]. The technique used here [10] improves the system performance [9], where the phase and amplitude of the single sidebands were more matched. The proposed approach decreased the maximum crosstalk level between the different MIMO channels as compared to transporting the same signals by using SCM technique. In addition, it could be applied to work with existing commercially available ROF systems, which were designed to carry just SISO radio signals.

Recently, three wireless 16-QAM MIMO signals were proposed to be transmitted over a 20 km SMF using the optical single-sideband frequency translation (OSSB-FT) technique (which is considered as OFU technique) [11]. These wireless MIMO signals were modulated using the carrier frequency of 2.44 GHz and optically modulated using the optical double-sideband (ODSB) modulation scheme. The ODSB modulation scheme is affected by the dispersion effects of the fiber segment. The fiber chromatic dispersion also increases directly proportional with increasing radio frequency (RF) modulating frequency [1]. In terms of the spectral efficiency, the ODSB modulation scheme is not attractive. The proposed communication system achieved a bit error rate (BER) of 10^−5^.

In this paper, the OFU technique is proposed to solve the problem of wireless MIMO signals transmission over fiber, since it does not need low-frequency local oscillators (LOs) at the transmitter and the receiver as compared to [10]. The crosstalk is highly suppressed between the different wireless MIMO signals with the same carrier frequency, since each wireless MIMO signal is carried on specific optical wavelength. By using the OFU technique, one optical dual-arm modulator (DAM) is derived by one optical source to produce multiple wavelengths which convey multiple wireless MIMO signals over the optical fiber. The FiWi system based on the new approach can also support the wavelength reuse technique, so one optical source is enough to generate the optical carrier which is reused at the AP as uplink wavelength, and multiple wavelengths, which convey multiple wireless MIMO signals over the SMF [12]. The principles and the simulation design of the OFU technique to transport wireless MIMO signals over fiber are discussed in Section 3.

The novel OFU approach is used to transmit three wireless MIMO signals over a 20 km SMF. The OFU technique exploits one optical source to produce multiple wavelengths by delivering it to a DAM. The parameters of the DAM are adjusted to produce number of wavelengths according to the number of the wireless MIMO signals. The wireless MIMO signals are then optically modulated by optical intensity modulators separately using the produced optical carriers from the OFU process. All these optical modulators are LiNbO_3_ Mach-Zehnder modulators (LN-MZMs). Each wireless MIMO signal is with a 2.4 GHz or 5 GHz carrier frequency, 1 Gb/s data rate, and 16-QAM. The crosstalk between the wireless MIMO signals is highly suppressed, since each wireless MIMO signal is carried on a specific optical wavelength. The system performance is evaluated in terms of BER, error vector magnitude (EVM), and eye diagrams for different RF carriers, optical link distances, and channel spacings. The novel technique provides a spectral efficient and reliable FiWi system.

This paper is organized as follows.  Section 2 outlines the operation of the OFU technique. Principles and design of the proposed system are demonstrated in Section 3. In Section 4, the mathematical model of the proposed system illustrates how the OFU approach operates in the proposed system.  Section 5 analyzes and discusses the system performance.  Section 6 suggests how the proposed approach can be extended to transport a higher number of wireless MIMO signals. Finally, conclusions are given in Section 7.

## 2. Optical Frequency Upconversion Technique

OFU technique is a prime technique in many fields of optical communication. External frequency modulators such as LN-MZMs can be used as a light-wave frequency upconverter in fiber optics [13]. The LN-MZM is a DAM which can be used as an optical frequency upconverter when its dual-arms are supplied by a sinusoidal RF signal. The LN-MZM is also used as an optical modulator for digital base-band signals or modulated RF signals, when these signals drive its dual-arms. For broadband communication applications, external LN-MZMs provide broadband operation and minimize the dispersion effects. Moreover, the external LN-MZMs offer high stability, very low bias-voltage drift rates, and bias-free devices [14, 15]. The frequency conversion efficiency of the LN-MZMs can be increased by using low values of half-wave voltage (*V*_*π*_).

In this study, the OFU technique is proposed to generate multiple optical carriers which are used to modulate multiple wireless signals separately at many optical external intensity modulators (IMs). The modulated optical signals can then be multiplexed together to the optical fiber, since they have no overlapping adjacent spectral bands. The DAM is set to generate first-order signal component (at the center sinusoidal RF frequency) and other higher-order modulated components around it. The higher-order components are neglected, since they have small amplitude compared to the lower-order components. In this approach, the WDM interleaver (WDM IL) is used after the DAM to separate the generated dominant wavelengths [16].

**Figure 2:**
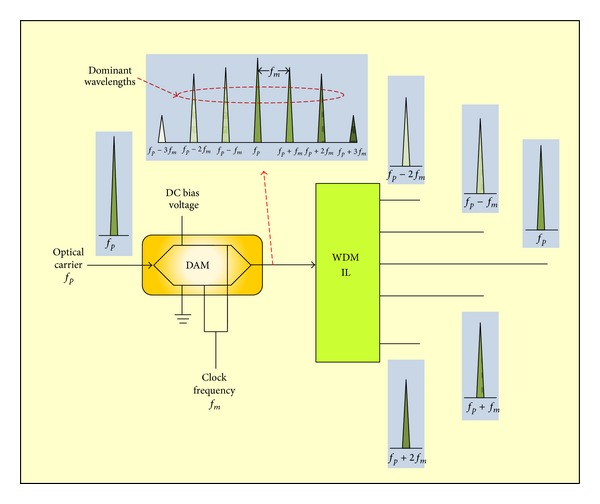
Generation of multiple wavelengths using OFU technique.

Generation of multiple wavelengths from one laser diode (LD) using OFU technique is illustrated in Figure 2. One optical source LD with optical carrier frequency *f*_*p*_ supplies a DAM which is driven by a sinusoidal clock frequency *f*_*m*_ (RF modulating frequency). The DAM is adjusted to generate multiple frequency components: first-order component with the center optical carrier frequency *f*_*p*_ and upper and lower single sidebands components around the center frequency. The lower single sideband components have the optical frequencies (*f*_*p*_ − *f*_*m*_, *f*_*p*_ − 2*f*_*m*_, *f*_*p*_ − 3*f*_*m*_, etc.). At the output, the upper single sideband components will have the optical frequencies (*f*_*p*_ + *f*_*m*_, *f*_*p*_ + 2*f*_*m*_, *f*_*p*_ + 3*f*_*m*_, etc.). From Figure 2, there are a number (five) of frequency components exceeding the other higher-order components which have small magnitudes as compared to their magnitudes. These frequency components are called dominant wavelengths or frequencies which are interleaved separately by using WDM IL. The channel frequency space (or wavelength interleave) between the generated wavelengths is *f*_*m*_. The dominant wavelengths will be used as downlink wavelengths to convey the multiple wireless MIMO signals over optical fiber.

## 3. Principles and Design of the Proposed System

**Figure 3:**
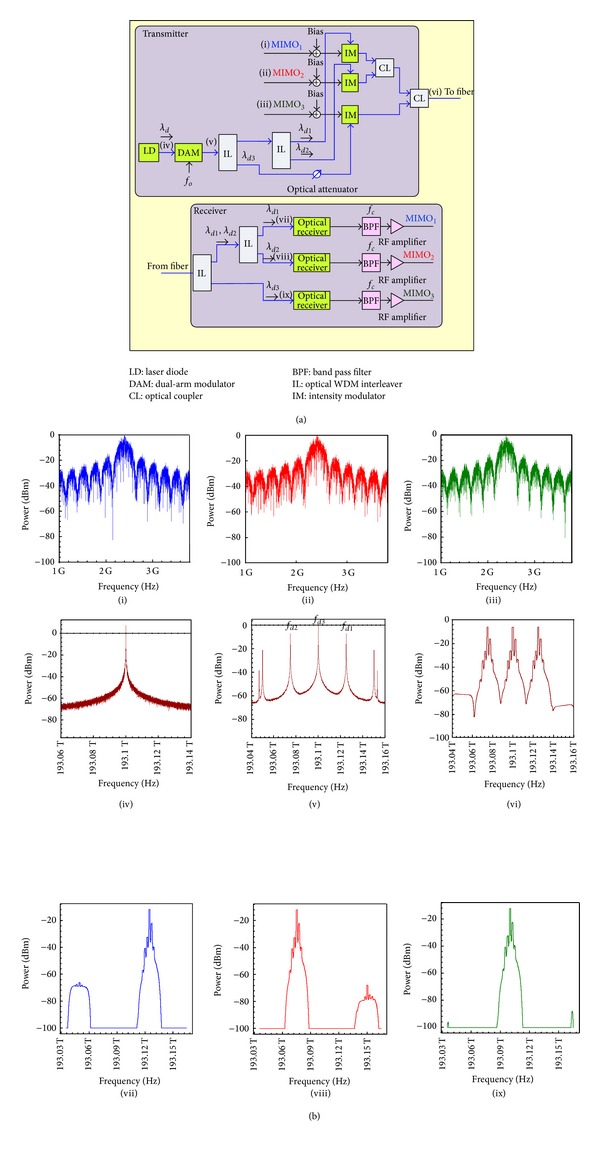
Transport of wireless MIMO signals over optical fiber using the OFU technique: (a) block diagram of the proposed technique and (b) power spectra of the signals according to the indicated insets.

The block diagram of the OFU technique, for transmission of three wireless MIMO signals over a single optical fiber, is shown in Figure 3(a). At the transmitter, three wireless MIMO signals MIMO_1_, MIMO_2_, and MIMO_3_ are generated and modulated using M-QAM at the same carrier frequency *f*_*c*_ = 2.4 GHz. The spectra of these three wireless signals are shown in Figure 3(b) in the insets ((i)–(iii)). A DAM, with the ODSB modulation technique, is used to generate three downlink wavelengths from one LD with a wavelength *λ*_*d*_ = 1552.52 nm (193.100 THz) as shown in Figure 3(b) the inset (iv). The three generated downlink wavelengths are shown in Figure 3(b) as the inset (v). Two ILs are used after the DAM to separate the three downlink wavelengths which are the two single-sideband wavelengths {*λ*_*d*__1_ = 1552.32 nm (193.125 THz), and *λ*_*d*__2_ = 1552.73 nm (193.075 THz)}, and the optical carrier frequency *λ*_*d*__3_ = 1552.52 nm (193.100 THz). The channel spacing between these wavelengths Δ*λ* equals the frequency of the sinusoidal clock *f*_*o*_ = 25 GHz (0.2 nm) which is used in the DAM. An optical attenuator is used in the path of the downlink wavelength *λ*_*d*__3_ to equilibrate its power with the generated power from the other downlink wavelengths *λ*_*d*__1_ and *λ*_*d*__2_.

The downlink wavelengths *λ*_*d*__1_, *λ*_*d*__2_, and *λ*_*d*__3_ are used to modulate the three wireless MIMO signals MIMO_1_, MIMO_2_, and MIMO_3_ by external IMs, respectively. The wireless MIMO signals are firstly biased to be compatible with the nature of the optical signals and then optically modulated by the IMs. These IMs use the OSSB+C modulation scheme to optimize the system performance against the fiber dispersion effect. The three modulated optical signals with the downlink wavelengths *λ*_*d*__1_, *λ*_*d*__2_, and *λ*_*d*__3_ are coupled together, as shown in Figure 3(b) inset (vi), and then propagated along a 20 km SMF with attenuation of 0.2 dB/km and dispersion coefficient of 17 ps/nm/km.

The receiver receives the optical downstream, and then interleaves it into three modulated optical signals with the wavelengths *λ*_*d*__1_, *λ*_*d*__2_, and *λ*_*d*__3_, as shown in Figure 3(b) in the insets ((vii)–(ix)). The receiver then downconverts the three modulated optical signals directly to the suitable electrical signals by using an optical receiver for each signal. The electrical signals are then band-pass filtered according to the allocated RF carrier frequency *f*_*c*_ = 2.4 GHz by using bandpass filters (BPFs) to get the wireless MIMO signals MIMO_1_, MIMO_2_, and MIMO_3_. The crosstalk between the received wireless MIMO signals which have the same frequency is highly suppressed, because each signal is carried on an independent wavelength with a large channel spacing (25 GHz) as compared to the carrier frequency (2.4 GHz). In this simulation, the PIN photodiodes with power sensitivity of −20 dBm are used in the optical receivers.

In the simulation design, the OFU technique is used to transport three wireless MIMO signals with the same RF carrier frequency of 2.4 GHz or 5 GHz over fiber. The wireless MIMO signals are modulated by using 16-QAM modulation to investigate the performance of this ROF system at different access distances and different wavelength interleaves.

## 4. Mathematical Model of the Proposed System

The optical field of the output signal *E*_out_(*t*) from the DAM can be expressed as [17, 18]:

(1) Eout(t)=αEin(t){(1−γ)e(jπv1(t)/VπRF+jπVb1/VπDC)+γe(jπv2(t)/VπRF+jπVb2/VπDC)}.

Here *E*_in_(*t*) is the input optical signal to the DAM from the LD. *v*_1_(*t*) and *v*_2_(*t*) are the RF modulating electrical voltage with the carrier frequency *f*_*m*_ = *ω*_*m*_/2*π*. *V*_*b*1_ and *V*_*b*2_ are the DC bias voltages applied to the arms of the DAM. *V*_*π*RF_ and *V*_*π*DC_ are the switching RF and switching bias voltages, respectively.

The parameter *α* is given by

(2) $$\begin{array}{c} \alpha =10^{-(\Omega /20)}. \end{array}$$

Here *Ω* is the insertion loss in dB (It is assigned as 5 dB in this design). *γ* designates the power splitting (combining) ratio of arm two for the input (output, resp.) *Y*-branch waveguide. *γ* is given by

(3) $$\begin{array}{c} \gamma =\frac{\left(1-1/\sqrt{\varepsilon_{r}}\right)}{2}, \end{array}$$

where *ε*_*r*_ = 10^(Extinction  Ratio/10)^ = 100, so *γ* ≈ 1/2 in this work.

In the simulation design, the values of *V*_*π*RF_ and *V*_*π*DC_ are set to 4 V, and the bias voltages of *V*_*b*1_ and *V*_*b*2_ are assigned as −1 V and 1 V, respectively. In addition, the generated optical signal from the LD can be expressed as *E*_in_(*t*) = *E*_*p*_ *e* ^*jω*_*p*_*t*^ and the modulating electrical signals can be expressed as *v*_1_(*t*) = −*v*_2_(*t*) = cos⁡(*ω*_*m*_ *t*). So (1) is rearranged as

(4) Eout(t)=12αe−j(π/4)Ein(t)(ej(π/4)v1(t)+jej(π/4)v2(t))=12αe−j(π/4)Epejωpt(ej(π/4)cos⁡ωmt+je−j(π/4)cos⁡ωmt).

From the Jacobi-Anger expansion [19],

(5) $$\begin{array}{c} e^{jm_{h}\cos\phi }=\sum_{n=-\infty }^{\infty }j^{n}J_{n}\left(m_{h}\right)e^{jn\phi }, \end{array}$$

where *J*_*n*_(*m*_*h*_) is the *n*-order Bessel function of the complex parameter *m*_*h*_.

The parameter *m*_*h*_ is called modulation index.

Therefore,

(6) ej(π/4)cos⁡ωmt=∑∞n=−∞jnJn(π4)ejnωmt=−jJ−1(π4)e−jωmt+J0(π4)+jJ1(π4)ejωmt,

where the values of *J*_*n*_(*π*/4) are neglected for *n* = ±2, ±3,…, ±*∞*, because of their too small values.

Also,

(7) e−j(π/4)cos⁡ωmt=ej(π/4)cos⁡(ωmt+π)=jJ−1(π4)e−jωmt+J0(π4)−jJ1(π4)ejωmt.

Since *J*_−*n*_(*z*) = (−1)^*n*^ *J*_*n*_(*z*) for integer value *n* [19], so *J*_−1_(*π*/4) = −*J*_1_(*π*/4). The expression of the output optical signal *E*_out_(*t*) is then simplified as

(8) Eout(t)=12α(1+j)e−j(π/4)Ep ×{J0(π4)ejωpt+J1(π4)[ej(ωp+ωm)t+ej(ωp−ωm)t]}=12αEp×{J0(π4)ejωpt+J1(π4)[ej(ωp+ωm)t+ej(ωp−ωm)t]}.

So the output signal can be expressed as

(9) Eout(t)=K1×ej(ωp+ωm)t +K2×ej(ωp−ωm)t+K3×ejωpt,

where *K*_1_, *K*_2_, and *K*_3_ are constants according to (8).

This signal is delivered to optical ILs to separate the three downlink optical carriers *f*_*d*1_ = *f*_*p*_ + *f*_*m*_  (*λ*_*d*1_), *f*_*d*2_ = *f*_*p*_ − *f*_*m*_  (*λ*_*d*2_), and *f*_*d*3_ = *f*_*p*_  (*λ*_*d*3_). Three wireless MIMO signals *M*_1_(*t*), *M*_2_(*t*), and *M*_3_(*t*) are OSSB+C modulated by these the three optical carriers *f*_*d*1_, *f*_*d*2_, and *f*_*d*3_, respectively, using three IMs as shown in Figure 3(a). The three MIMO signals have different QAM data stream at the same carrier RF of *f*_*c*_ = *ω*_*c*_/2*π*.

The modulated OSSB+C optical signal at each IM can be written as [18]

(10) $$\begin{array}{c} E_{\text{SSB}i}\left(t\right)\approx C_{i}e^{j\omega_{di}t}+M_{i}\left(t\right)e^{j(\omega_{d}+\omega_{c})t}, \end{array}$$

where *C*_*i*_ is a constant, *ω*_*di*_ = 2*πf*_*di*_ is the optical downlink carrier, *M*_*i*_ is the *i*th wireless MIMO signal with RF carrier frequency of *f*_*c*_, and *i* is the index of MIMO signal (*i* = 1,2, or 3). The three modulated optical signals by the optical wavelengths (*λ*_*d*__1_, *λ*_*d*__2_, and *λ*_*d*__3_) are combined into a single optical fiber. So the input optical signal to the optical fiber is given by

(11) Ein  fiber(t)≈C1ej(ωp+ωm)t+M1(t)ej(ωp+ωm+ωc)t +C2ej(ωp−ωm)t+M2(t)ej(ωp−ωm+ωc)t +C3ejωpt+M3(t)ej(ωp+ωc)t.

This signal propagates along an SMF with the propagation constant of *β*(*ω*) and attenuation magnitude *α*_*f*_, where *ω* is the angular frequency. So the output lightwave at the end of the SMF with length of *z* can be approximated as [20]

**Figure 4:**
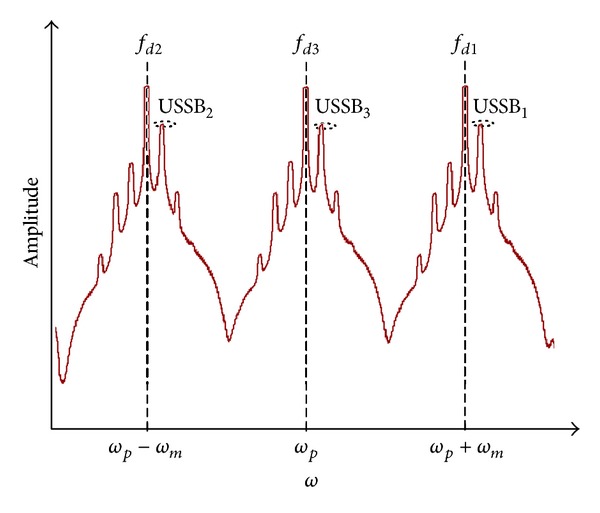
The propagated lightwave signal over the optical fiber.

(12) Eout  fiber(z,t)∝e−αfz{C1ej[(ωp+ωm)t+β(ωp+ωm)z]+M1(t−td1)×ej[(ωp+ωm+ωc)t+β(ωp+ωm+ωc)z]+C2ej[(ωp−ωm)t+β(ωp−ωm)z]+M2(t−td2)×ej[(ωp−ωm+ωc)t+β(ωp−ωm+ωc)z]+C3ej[ωpt+β(ωp)z]+M3(t−td3)ej[(ωp+ωc)t+β(ωp+ωc)z]},

where *t*_*di*_ (*i* = 1,2, or 3) is the time delay of the *i*th optical downlink signal. The time delay is calculated by the first derivative of *β*(*ω*), since *t*_*di*_ = *β*′(*ω*_*di*_ + *ω*_*c*_), and *ω*_*i*_ is the *i*th optical downlink carrier frequency. The output lightwave at the end of fiber is considered as three optical signals with different downlink frequencies of *ω*_*p*_ + *ω*_*m*_, *ω*_*p*_ − *ω*_*m*_, and *ω*_*p*_ which convey the three wireless MIMO signals in their upper single sidebands (USSBs) as shown in Figure 4.

The optical receiver receives the transmitted optical signals and separates them according to their downlink wavelengths by using optical ILs as shown in Figure 3. Each optical downlink signal is then directly detected by a photodetector (PD), so the photocurrent for each detected MIMO signal can be written as the following equation according to the square-law PD [18]:

(13) Ii(z,t)=ρ|Ei(z,t)|2=ρEi(z,t)×Ei∗(z,t)∝ρe−2αfz×{Ciej[ωdit+β(ωdi)z]+Mi(t−tdi)ej[(ωdi+ωc)t+β(ωdi+ωc)z]} ×{Cie−j[ωdit+β(ωdi)z]+Mi(t−tdi)e−j[(ωdi+ωc)t+β(ωdi+ωc)z]}Ii(z,t)∝ρe−2αfz ×{Ci2+Mi2(t−tdi)+2CiMi(t−tdi)cos⁡(ωc(t+βz))},

where *ρ* is the responsivity of the photodetector.

According to (13), the photocurrent is comprised of the DC component and the RF component at *ω*_*c*_ after transmission. The detected signal is then passed through BPF with a center frequency of *f*_*c*_, so the DC component is removed. Each detected wireless MIMO signal with the carrier frequency *f*_*c*_ is directly amplified and propagated by using MIMO antenna technique through wireless channel. The wireless end-user will receive the three MIMO signals and demodulate them using the suitable QAM demodulation and MIMO decoding techniques.

## 5. System Performance Evaluation

**Figure 5:**
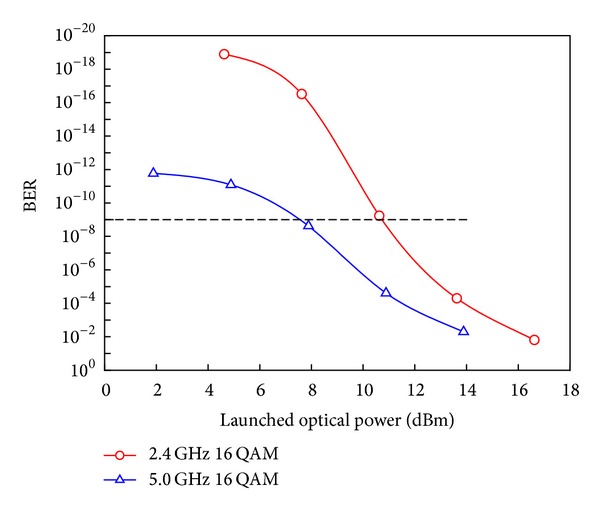
The fiber nonlinearity effect on the system performance.

In this work, the communication system is designed to provide a data rate of 1 Gb/s for each 16-QAM wireless MIMO signal. Figure 3 in the inset (vi) shows the input optical power to the optical fiber, where the three modulated optical signals with the downlink wavelengths *λ*_*d*__1_, *λ*_*d*__2_, and *λ*_*d*__3_ are coupled to propagate through 20 km optical fiber. The total input power of the three optical signals is 1.626 dBm according to the simulation calculations for both RF carrier frequencies of 2.4 GHz and 5 GHz. This power is suitable to be launched to avoid the nonlinear effects along the optical link.  Figure 5 shows the system performance at different launched optical powers. Nonlinearity of the fiber negatively affects the system performance when the launched optical powers are greater than 10.8 dBm and 7.9 dBm at the carrier frequencies of 2.4 GHz and 5.0 GHz, respectively.

**Figure 6:**
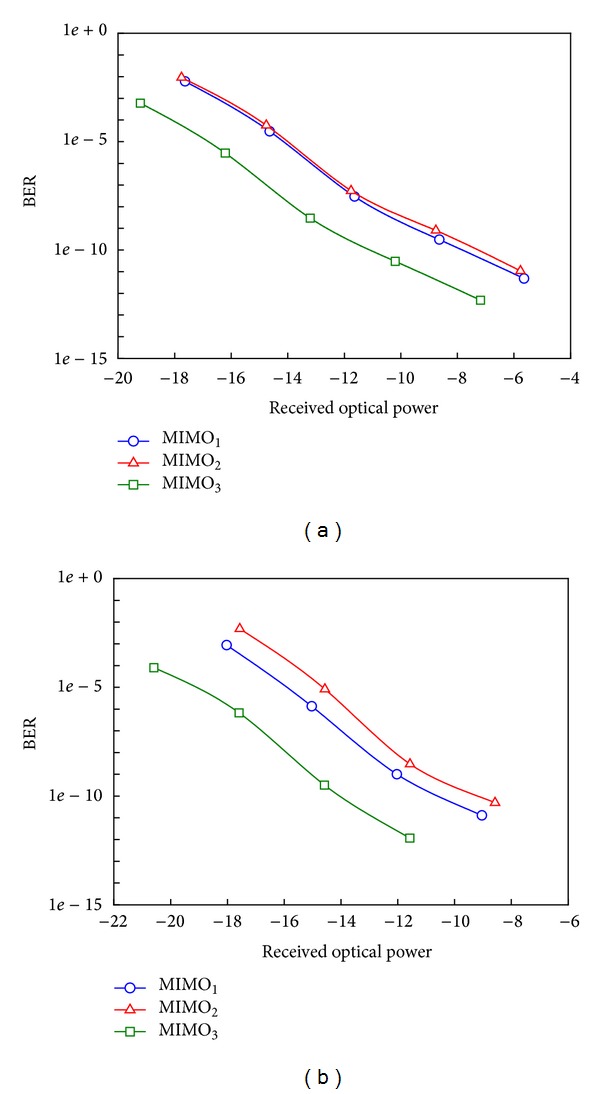
The BER performance versus received optical power at the carrier frequencies: (a) 2.4 GHz and (b) 5 GHz.

To evaluate the performance of the proposed technique, Figures 6(a) and 6(b) show the BER performance versus the received optical power at the receiver for the three wireless MIMO signals (MIMO_1_, MIMO_2_, and MIMO_3_) at the carrier frequencies 2.4 GHz and 5 GHz, respectively. The power sensitivity differences of the receivers for the three MIMO signals are small, especially between the two MIMO signals (MIMO_1_ and MIMO_2_). The maximum power penalties of 3.47 dB and 4 dB are recorded at BER of 10^−9^ for the carrier frequencies of 2.4 GHz and 5 GHz, receptively.

**Figure 7:**
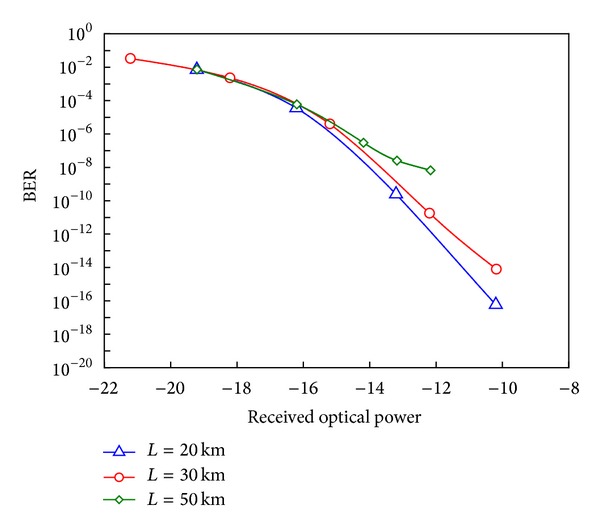
The system performance at different lengths of the optical fiber.

Figure 7 shows the system performance at three different fiber lengths (20 km, 30 km, and 50 km) of the optical fiber. In the proposed system, the fiber length of 50 km has slight effect on the performance of the transmitted optical signals which carry the wireless MIMO signals. The system performance deteriorates progressively when the access distance becomes longer than 50 km.

**Figure 8:**
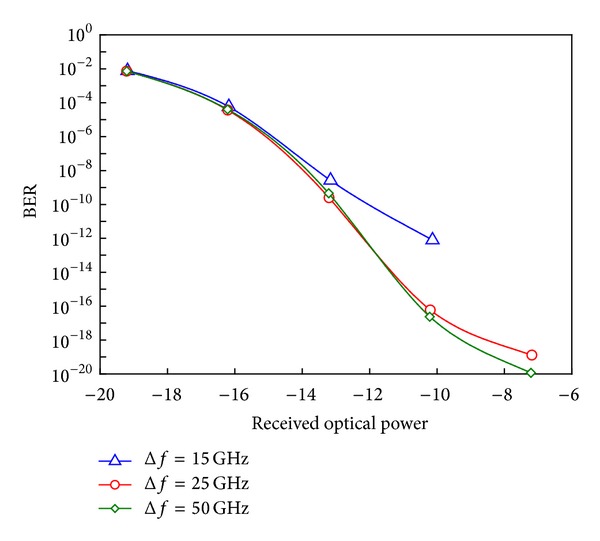
The system performance at different wavelength interleaves.

In addition, the system performance is analyzed by using different wavelength interleaves between the optical carrier frequency (or RF clock frequency *f*_*o*_).  Figure 8 shows the system performance at different wavelength interleaves (Δ*f* = 15, 25, and 50 GHz which are compatible with Δ*λ* = 0.12, 0.2, and 0.4 nm, resp.). When the wavelength interleaves are smaller than 15 GHz, the system performance will degrade and the error floor clearly appears.

**Figure 9:**
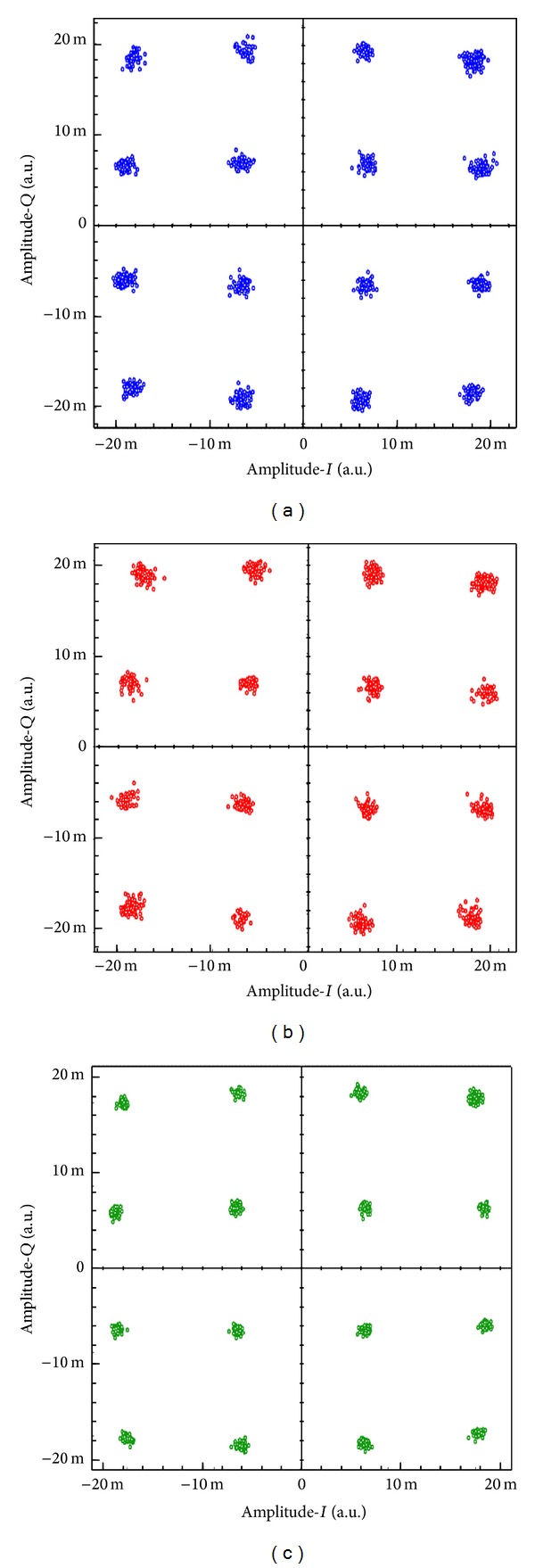
Constellation diagrams of the demodulated 16-QAM MIMO signals (a) MIMO_1_, (b) MIMO_2_, and (c) MIMO_3_.

Figures 9(a)–9(c) show 1 Gb/s 16-QAM constellation diagrams for the received MIMO signals MIMO_1_, MIMO_2_, and MIMO_3_, respectively at 2.4 GHz. Clear scatter-plots are achieved at EVM values of −20.8780 dB, −20.2873 dB, and −21.2961 dB for MIMO_1_, MIMO_2_, and MIMO_3_, respectively. So the proposed technique has achieved a good performance of transmitting wireless MIMO signals over the optical fiber at the carrier frequencies 2.4 GHz and 5 GHz. The EVMs are calculated considering the following equation [21]:

(14) $$\begin{array}{c} \text{EVM}\left(\text{dB}\right)=10\cdot \text{lo}{\text{g}}_{10}\left[\frac{\sum_{k=1}^{M}{\left|S_{tx,k}-S_{rx,k}\right|}^{2}}{\sum_{k=1}^{M}{\left|S_{tx,k}\right|}^{2}}\right], \end{array}$$

where EVM is the value of the difference between a collection of received symbols and transmitted or ideal symbols, *S*_*tx*,*k*_ is the corresponding transmitted symbol of the constellation associated with the *k*th symbol, *S*_*rx*,*k*_ is the received symbol associated with *S*_*tx*,*k*_, and *M* is the number of the symbols for the* inphase*-*quadrature *constellation.

**Figure 10:**
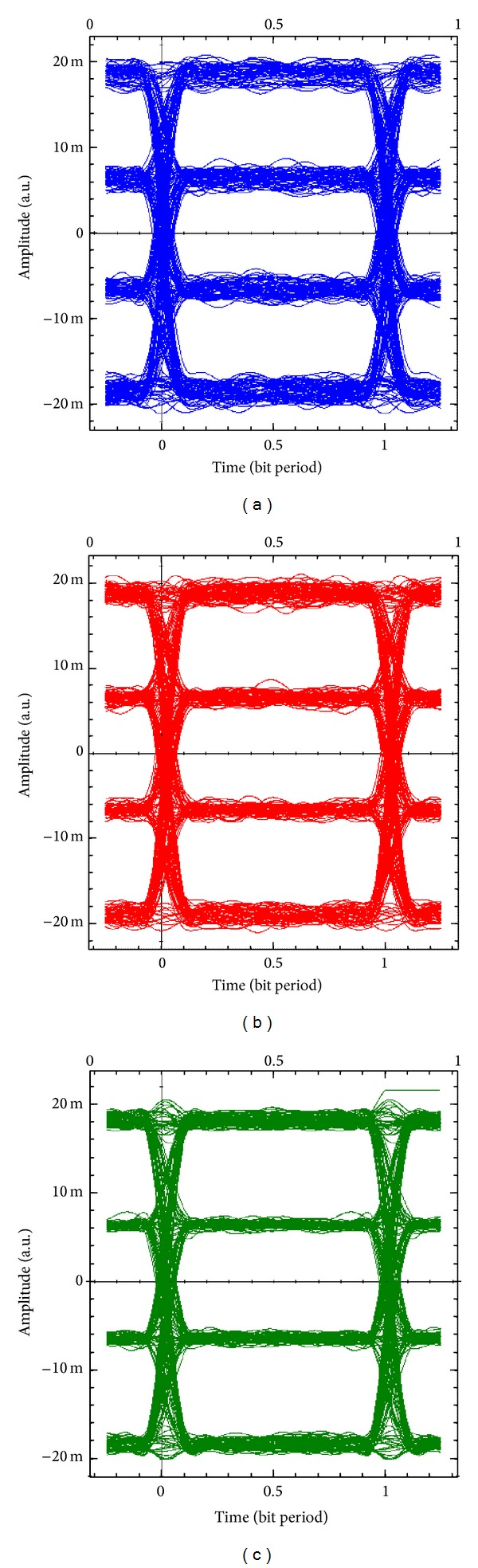
Eye diagrams of the *I*-branch of the demodulated 16 QAM baseband signals for (a) MIMO_1_, (b) MIMO_2_, and (c) MIMO_3_.

**Figure 11:**
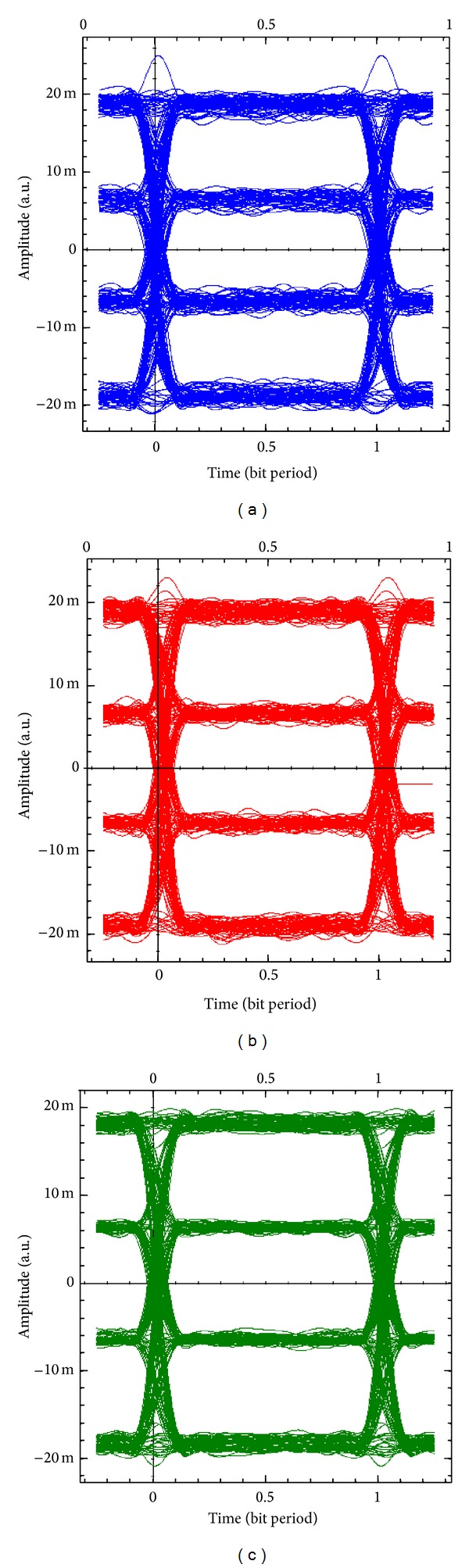
Eye diagrams of the *Q*-branch of the demodulated 16 QAM baseband signals for (a) MIMO_1_, (b) MIMO_2_, and (c) MIMO_3_.

Figures 10(a)–10(c) show the eye diagrams of the *I*-branch of the received 16-QAM baseband signals for MIMO_1_, MIMO_2_, and MIMO_3_, respectively. Also Figures 11(a)–11(c) show the eye diagrams of the *Q*-branch of the received 16-QAM baseband signals for MIMO_1_, MIMO_2_, and MIMO_3_, respectively. The eye diagrams of both *I*-branch and *Q*-branch of the received wireless MIMO signals at the receiver show slight differences and good quality communication system at a BER around of 10^−9^. The BER are calculated according to (15) [22]:

(15) BER≈(1−Q−1)log2Q∗ε,ε=erfc⁡[12·3·log2Q(Q2−1)·2(k·EVMrms)2·log2M],k=|Stx,max⁡|∑i=1M(|Stx,i|/M),

where *Q* is the number of signal levels within each branch of the constellation diagram, log_2_ *M* is the amount of bits encoded into one QAM symbol, and *k* is a modulation format-dependent factor giving the relationship between maximum field magnitude and average overall *M* field magnitudes defined by the constellation diagram for the chosen modulation format. This factor is calculated according to (15) to be $6/\left(\sqrt{5}+2\right)$ for 16-QAM. The *S*_*tx*,*i*_ is the ideal transmitted field vector, and *S*_*tx*,max⁡_ is the field vector of the outermost constellation point. In this paper, the performance of EVM and the BER is evaluated for the 16-QAM MIMO signals without using forward error correction (FEC) techniques.

## 6. Transmission of More Wireless MIMO Signals over Optical Fiber

**Figure 12:**
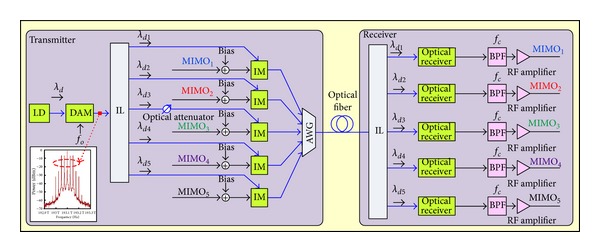
Transmission of five wireless MIMO signals over fiber using the novel approach.

Figure 12 shows the proposed OFU technique to transmit five wireless MIMO signals over fiber. At the OLT, the DAM is injected by LD with the wavelength *λ*_*d*_. The DAM is a LN-MZM. Adjusting the parameters of the DAM to suitable values can generate multiple wavelengths. The dominant wavelengths are considered, and the remaining outside wavelengths are neglected because of their very small magnitudes. The OLT allocates five downlink wavelengths (*λ*_*d*1_, *λ*_*d*2_, *λ*_*d*3_, *λ*_*d*4_, and *λ*_*d*5_) which are used for downstream modulation. To generate five dominant wavelengths, the values of DAM parameters are configured as −0.5 V, 0.5 V DC bias voltages are applied to, respectively, first and second arms of the LN-MZM, the RF clock voltage with frequency of *f*_*o*_ drives to the DAM, and the DC and RF switching voltages are set to 4 V and 2 V, respectively. The wavelength interleaves between the five generated wavelengths which are equal to the frequency of the sinusoidal clock *f*_*o*_. The power magnitudes of the five wavelengths are approximately equal and the center wavelength *λ*_*d*3_ has maximum value. The difference between this and the others is around 6 dB. To get balanced power magnitudes, an optical attenuator is used in path of the center wavelength after IL, as shown in Figure 12. Each generated wavelength modulates the MIMO signal by using IM. The five modulated optical signals propagate along the same optical fiber.

The receiver receives the optical downstream and then interleaves it into the five modulated optical signals with the wavelength *λ*_*d*1_, *λ*_*d*2_, *λ*_*d*3_, *λ*_*d*4_, and *λ*_*d*5_ as shown in Figure 12. The receiver then downconverts the five modulated optical signals directly to the suitable electrical signals by using an optical receiver for each signal. The electrical signals are then band-pass filtered according to the allocated RF carrier frequency *f*_*c*_ by using BPFs to get the original five wireless MIMO signals MIMO_1_, MIMO_2_, MIMO_3_, MIMO_4_, and MIMO_5_.

## 7. Conclusions

The novel OFU technique is proposed to solve the problem of wireless MIMO signals transmission over a single optical fiber. Three wireless 16-QAM MIMO signals have been transmitted over a 20 km SMF using the OFU technique. These wireless MIMO signals were modulated using the carrier frequency of 2.4 GHz or 5 GHz at data rate of 1 Gb/s for each signal. The physical layer performance has been reported in terms of the BER at different RF carrier frequencies, different access distances, and different wavelength interleaves. In addition, the EVM and the eye diagrams are analyzed in this study.

The proposed approach highly suppressed the crosstalk between different MIMO signals with the same RF carrier frequency, since each MIMO signal is carried on a specific optical wavelength. While the ESSB-FT technique [10] requires a number of low-frequency LOs and electrical BPFs at the transmitter and the receiver, the OFU technique does not require low-frequency LOs at the transmitter and the receiver or electrical BPFs at the transmitter. Less number of electrical BPFs is required at the receiver in the proposed technique. However, a number of PDs are required at the receiver which is equal to the number of MIMO signals. The proposed system supports many wavelengths for carrying multiple wireless MIMO signals over the fiber using single LD. The novel technique provides a spectral efficient and reliable FiWi system.
